# Development of a child and family centred outcome measure for children and young people with life-limiting and life-threatening conditions: progress to date on the Children’s Palliative Care Outcome Scale (C-POS:UK)

**DOI:** 10.1177/26323524241303537

**Published:** 2024-12-17

**Authors:** Debbie Braybrook, Lucy Coombes, Daney Harðardóttir, Hannah M. Scott, Katherine Bristowe, Clare Ellis-Smith, Anna Roach, Christina Ramsenthaler, Myra Bluebond-Langner, Julia Downing, Fliss E. M. Murtagh, Lorna K. Fraser, Richard Harding

**Affiliations:** Cicely Saunders Institute of Palliative Care, Policy & Rehabilitation, Florence Nightingale Faculty of Nursing Midwifery & Palliative Care, King’s College London, Bessemer Road, London SE5 9PJ, UK; Cicely Saunders Institute of Palliative Care, Policy & Rehabilitation, Florence Nightingale Faculty of Nursing Midwifery & Palliative Care, King’s College London, London, UK; Royal Marsden NHS Foundation Trust, London, UK; Cicely Saunders Institute of Palliative Care, Policy & Rehabilitation, Florence Nightingale Faculty of Nursing Midwifery & Palliative Care, King’s College London, London, UK; Cicely Saunders Institute of Palliative Care, Policy & Rehabilitation, Florence Nightingale Faculty of Nursing Midwifery & Palliative Care, King’s College London, London, UK; Cicely Saunders Institute of Palliative Care, Policy & Rehabilitation, Florence Nightingale Faculty of Nursing Midwifery & Palliative Care, King’s College London, London, UK; Cicely Saunders Institute of Palliative Care, Policy & Rehabilitation, Florence Nightingale Faculty of Nursing Midwifery & Palliative Care, King’s College London, London, UK; Faculty of Population Health Sciences, Great Ormond Street Institute of Child Health, London, UK; Cicely Saunders Institute of Palliative Care, Policy & Rehabilitation, Florence Nightingale Faculty of Nursing Midwifery & Palliative Care, King’s College London, London, UK; Wolfson Palliative Care Research Centre, Hull York Medical School, Hull, UK; Department of Health Sciences, Zurich University of Applied Sciences, Winterthur, Switzerland; Louis Dundas Centre for Children’s Palliative Care, Great Ormond Street Institute of Child Health, University College London, London, UK; Cicely Saunders Institute of Palliative Care, Policy & Rehabilitation, Florence Nightingale Faculty of Nursing Midwifery & Palliative Care, King’s College London, London, UK; International Children’s Palliative Care Network, Kampala, Uganda; Wolfson Palliative Care Research Centre, Hull York Medical School, Hull, UK; Cicely Saunders Institute of Palliative Care, Policy & Rehabilitation, Florence Nightingale Faculty of Nursing Midwifery & Palliative Care, King’s College London, London, UK; Cicely Saunders Institute of Palliative Care, Policy & Rehabilitation, Florence Nightingale Faculty of Nursing Midwifery & Palliative Care, King’s College London, London, UK

**Keywords:** outcome measure development, paediatrics, palliative care, patient-centred outcome measure

## Abstract

**Background:**

Development of a paediatric palliative care child and family centred outcome measure is a priority for health care professionals, researchers and advocates. It is methodologically challenging to develop a measure relevant for such a heterogenous population with complex needs. Involving children in measuring development is vital.

**Objective:**

To develop C-POS:UK (Children’s Palliative Care Outcome Scale, UK), a person-centred outcome measure (PCOM) for children with life-limiting conditions and their families, and to test its psychometric properties.

**Design:**

Sequential mixed-methods approach to PCOM development, guided by Rothrock’s measure development process and COnsensus-based Standards for the selection of health Measurement Instruments (COSMIN) methodology.

**Methods:**

(i) Qualitative interviews about priority symptoms and concerns, with embedded exploration of measure design for children with life-limiting conditions; (ii) systematic review of measure design for children; (iii) modified Delphi survey, and consultation with children, on priority items for new measure; (iv) expert item generation meeting to develop C-POS:UK; (v) cognitive testing to refine C-POS:UK; (vi) psychometric validation.

**Results:**

(i) 106 participants described physical, emotional/psychological, spiritual/existential, social and practical concerns. Measure design was discussed by 79 participants comprising preferred response format, recall period and measure administration for children with life-limiting conditions; (ii) systematic review highlighted need for: different versions of measure accounting for child’s developmental stage and cognitive ability; parent/carer involvement as proxies for very young children; and testing to clarify recall periods and response formats at different developmental stages; (iii) Delphi survey: 82 participants (in the first round), with a move towards consensus, but with some differing priorities in stakeholder groups: professionals prioritised physical symptoms, parents prioritised psychosocial and practical matters, while consulted children prioritised normality; (iv) 22 experts contributed to item generation meeting, resulting in five versions of C-POS:UK accounting for child’s developmental stage and cognitive ability, and proxy involvement; (v) 48 participants cognitively tested initial C-POS:UK, informing comprehension, comprehensiveness and acceptability; (vi) psychometric validation is ongoing.

**Conclusion:**

A sequential approach informed by Rothrock and COSMIN has supported development of the first version of C-POS:UK. Psychometric validation is underway and will be followed by implementation planning.

## Background

Worldwide, approximately 30% of the population is under 18 years old.^1^ Data suggests that the number of babies, children and young people (hereafter ‘children’) living with life-limiting or life-threatening conditions (hereafter ‘life-limiting’) is increasing.^2,3^ This can be partly attributed to advances in medicine and technology that have altered disease trajectories, meaning children are living longer with more complex needs and higher care dependency. Palliative care has the potential to ease the symptoms experienced by an estimated 21 million children worldwide who are living with such conditions, and there is increasing recognition of the benefits of the palliative approach to care.^4^ However, the differing rates of paediatric palliative care development and resources across the globe mean that needs are not being met.^2,5^

Outcome measurement offers an evidence-driven approach that can support the development of appropriate palliative services for children and families worldwide. Person-centred outcome measures (PCOMs) are powerful tools that promote higher-quality care by providing professionals with information to monitor patient health, and in turn, respond to their priority concerns. They may also be used for auditing and monitoring service provision, or by researchers seeking to understand care quality or the impact of interventions. PCOMs are either completed by the patient themselves (sometimes called patient-reported outcome measures, or PROMs) or by proxies who answer on behalf of the patient when they do not have the capacity to. Evidence from adult palliative care demonstrates that PCOMs promote improvements in patient-clinician communication and collaborative working, enabling professionals to better recognise symptoms and drive care that is congruent with patient priorities.^6,7^ A qualitative interview study with children with life-limiting conditions, their families and the professionals who care for them confirmed that a robustly developed and implemented palliative care PCOM for children is expected to bring similar benefits.^8^

Development of a measure for paediatric palliative care has been repeatedly identified as a research priority.^9–12^ An expert stakeholder workshop to plan the UK Children’s Palliative care Outcome Scale (C-POS:UK) proposal demonstrated that a child and family centred outcome measure (hereafter, PCOM) was considered a priority across clinical, research and advocacy groups.^13^ Paediatric palliative care nurses, clinicians, researchers and advocates from across the United Kingdom indicated the need for a PCOM for children with life-limiting conditions, covering children’s physical, psychological, social and spiritual needs, as described in the World Health Organization (WHO) definition of palliative care.^14^ Despite a clear need for a PCOM that considers the things that children with life-limiting conditions say are most important to them, measure development for this group has several challenges. There are a large number of conditions that would benefit from a palliative approach, meaning evidence to inform a PCOM that will be useful across the spectrum must be drawn from a varied population. A systematic review conducted by Namisango et al.^15^ demonstrated that almost 75% of evidence about priorities of care for children with life-limiting conditions comes from children with an oncological condition, their family or treating professionals. This is particularly pertinent given that in the United Kingdom, the most common diagnoses are for children with congenital, perinatal, neurological, respiratory and haematological conditions, in descending order.^3^ Furthermore, the heterogeneity in age and developmental delay in some children with life-limiting conditions complicates engagement. Thirty percent of studies included in the abovementioned systematic review did not include children but rather relied on parents and health workers to describe the health outcomes that mattered to children.^15^ This requires recognition that some children may not be able to participate due to their condition. While involving parents as proxies for children who cannot complete the measure is required, parents’ outcomes independent of the child were also viewed as important.

The challenging nature of developing a PCOM for and with children with life-limiting conditions has, until recently, remained unaddressed. A systematic review conducted by Coombes et al.^16^ demonstrated that the domains of generic outcome measures were not always relevant to children with life-limiting conditions, and disease-specific measures would not allow a comparison of outcomes between such a heterogeneous population.^16^ Since that review, a measure has been under development in sub-Saharan Africa and Belgium, with measurement properties informed by data from within Africa.^17–19^ Cultural validity is central to ensure that the most important contextually relevant outcomes for children and their families are being measured.

Wherever possible, children should have the opportunity to be active partners in their healthcare, not passive recipients.^20,21^ Paediatric palliative care experts recommended that special attention be given to ensuring that children could participate in measure development wherever practicable.^13,22^ As such, children should be given the opportunity to be engaged in the development of such a PCOM if they are able and choose to do so. In-depth exploration of the views of children with diverse life-limiting conditions, their families (including parents and siblings), and the professionals responsible for their care was highlighted as the next step in developing the PCOM.^13^

## Objectives

The overarching aim of the C-POS:UK study is to develop a PCOM that can be used by children with life-limiting conditions and their families, and to test its psychometric properties. The following objectives, presented in study phases, have been developed to achieve this aim.

### Phase I: Gathering input on measure concept and design

i. Determine priority symptoms and concerns, and care priorities of children with life-limiting conditions and their families; and to identify preferences for PCOM design among children with life-limiting conditions, and their families.
ii. Synthesis evidence on measure design and approaches needed to enable children to participate in valid and reliable self-reporting of their health outcomes.

### Phase II: Item generation

iii. Establish stakeholder consensus on items to include in the PCOM.
iv. Using data from objectives (i)–(iii), agree final items, how to ask and measure design aspects, then generate the draft C-POS:UK.

### Phase III: Item improvement

v. Establish comprehensibility, relevance, comprehensiveness and acceptability of the initial C-POS:UK within the target population.

### Phase IV: Initial psychometric validation (currently underway)

vi. Determine the psychometric properties of C-POS:UK for children facing life-limiting conditions and their families.

## Methods

### Design

Sequential mixed-methods study drawing on Rothrock et al.’s^23^ recommended measure development process and COnsensus-based Standards for the selection of health Measurement INstruments (COSMIN).^23–25^ The reporting of this study conforms to the guidelines for Good Reporting of A Mixed Methods Study^26^ (Supplemental File 1).

Mixed methods are essential to outcome measure development, as they enable the best method to be applied for progressive knowledge development, with each study feeding into one or more consecutive studies. The sequential stages of development of C-POS:UK described here comprise gathering input on the content and design of the measure (qualitative interview study of symptoms and care priorities, with embedded exploration of measure design for children with life-limiting conditions; systematic review of measure design of PCOMs for children), expert concept clarification and item generation (Modified ranking-style Delphi survey, and item generation meeting, with consultation with children), item improvement (cognitive interviewing), and initial psychometric validation (repeated observational questionnaire study).

**Figure 1.**
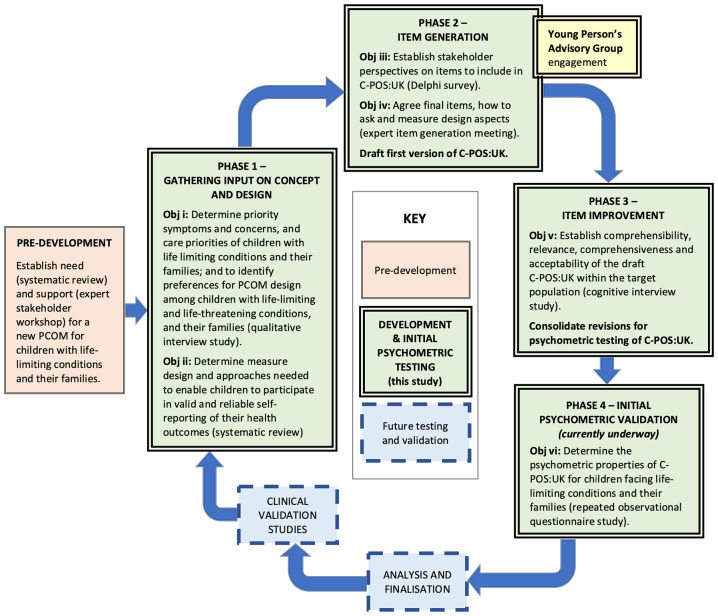
C-POS:UK (UK Children’s Palliative Care Outcome Scale) study mapped onto Rothrock’s measure development process.

Rothrock et al.^23^ provide a recommended mixed-methods process to follow when developing a new PCOM. This PCOM development process has been adapted for the C-POS:UK study (see Figure 1). The COSMIN criteria are not intended to support the development of a PCOM, but to evaluate the quality of existing measures.^23–25,27–34^ However, using these checklists in conjunction with Rothrock’s guidance can provide a robust design and reporting structure for PCOM development. COSMIN criteria and guidance were used to inform the C-POS:UK development. This includes the COSMIN guidelines for evaluating the ‘content validity’ of existing PCOMs,^24^ which entails ‘relevance’ (if items, along with their associated response options and recall periods, are relevant to the target population, construct of interest and context of use), ‘comprehensiveness’ (if the PCOM covers all relevant aspects of the construct to be measured) and ‘comprehensibility’ (if the items are understood by the target population).^32^ COSMIN guidelines also highlight ‘acceptability’ (willingness of patients to complete a measure)^24^ and ‘feasibility’ (ease of application of a PCOM in its intended context of use)^25^ as factors that require careful consideration in PCOMs.

The Rothrock development process and COSMIN guidelines were utilised together to inform the robust sequential mixed-methods design and reporting of C-POS:UK. Each study is reported in detail elsewhere,^35–39^ but summarised here to demonstrate the innovative approach taken to meet the study aim and objectives.

## Methods, by phase and objective

### Phase I: Gathering input on measure concept and design

i. Semi-structured qualitative interview study with children with any life-limiting condition (aged 5–17 years), their siblings (5–17 years old), parents/carers of a child <18 years old with a life-limiting condition, healthcare professionals with >6 months experience of providing care for children with life-limiting conditions, and those who decide which UK paediatric palliative care services to fund (hereafter ‘commissioners’).^35^ The topic guide was informed by the WHO definition of paediatric palliative care,^14^ and a systematic review of symptoms and concerns of children with life-limiting conditions.^15^ Participants were recruited across nine UK sites. Pseudonymised verbatim interview transcripts were analysed, deductively and inductively, using framework analysis informed by the WHO domains of palliative care for children.^14^
ii. Qualitative exploration of measure design preferences (recall period, response format, length and administration mode) among the target population.^38^ This objective was addressed within the qualitative interviews with children with life-limiting conditions, siblings and parents, described above. Participants were asked questions about response format, recall period and measure administration, and matters of relevance, comprehensibility, feasibility and acceptability, as described in COSMIN guidance on content validity.^24^ These data were analysed using framework analysis and the COSMIN guidance on content validity.^24^
iii. Systematic review to appraise evidence on recall period, response scale format, mode of administration and approaches needed to enable children ⩽ 18 years to report on their own health outcomes.^37^ The review was conducted and reported in accordance with PRISMA guidelines,^40^ and the quality of returned articles was assessed using QualSyst, in accordance with the focus on overall measure design. Narrative synthesis was developed from the results.^37^

### Phase II: Item generation

iii. Modified ranking-style Delphi survey,^36^ conducted and reported using Conducting and REporting DElphi Studies (CREDES) guidance.^41^ Key concepts identified in phase I and those identified in a systematic review by Namisango et al.^15^ for the African Palliative Care Association (APCA) African C-POS were presented in an online three-round Delphi ranking survey to parents and professionals with experience of caring for a child with a life-limiting condition.^36^

In the first round, a list of 42 items was presented to the expert stakeholder group. They ‘narrowed down’ the list by selecting the top 20 items to be included in C-POS:UK, and detailed any additional items that they believed to be missing from the list.

Results from the ‘narrowing down’ round were then reported back to round one participants in the first ‘ranking’ round. In ranking round 1, retained items were reported in random order to participating experts, who were then invited to rank them in order of priority for inclusion in C-POS:UK. In ranking round 2, items were reported in order of mean rank. Concordance was measured using Kendall’s *W* coefficient of concordance (interpreted as weak <0.5, moderate 0.5–0.7, strong >0.7) and ranking in the top 50%. The Delphi process would cease upon reaching a consensus (Kendall’s *W* >0.7). Ranking agreement between professional and parent was determined using Cohen’s kappa.^36^

Due to concerns about feasibility of the Delphi survey recruitment and consent processes for children, engagement of children was achieved through consultation. A Young Person’s Advisory Group (YPAG) was consulted on priority outcomes, to ensure children and young people’s perspectives were considered in the refinement of the C-POS:UK (see Figure 1). Younger children were asked to reach a group consensus on their top 10 outcomes, while older children reviewed and chose their top 10 outcomes independently.

iv. Half-day virtual item generation meeting was conducted with the C-POS:UK expert steering group, which includes parents bereaved of a child with life-limiting conditions health and social care professionals who care for children with life-limiting conditions, clinical academics and academics.^36^ The item generation meeting was guided by COSMIN guidelines which highlight the importance of expert involvement for content validity.^29^

Although COSMIN standards do not provide direct guidance on generating items for PCOMs, due to the focus on assessing pre-existing measures, the COSMIN criteria for assessing content validity was used to guide item generation.^24^ After presentation of the construct to be measured by C-POS:UK (informed by objective (i) results), priority items for inclusion (informed by objective (iv) results), measure design (informed by objectives (ii) and (iii) results) and suggested wording was presented and discussed.^36^

### Phase III: Item improvement

v. Cognitive testing^39^ of the initial C-POS:UK (developed using results from objectives (i)–(iv)) with children with any life-limiting condition (aged 5–17 years), and parents/carers of a child <18 years old with a life-limiting condition, reported using the Cognitive Interview Reporting Framework.^42^ Participants were recruited across 14 UK sites.

The appropriate version of the initial C-POS:UK was selected by parents and children, guided by the child developmental stage and cognitive ability.

Interviews were conducted using the ‘think aloud’ method, whereby participants were encouraged to describe their thought process while reading the questions and selecting their answers.^43,44^ A practice task was utilised to familiarise participants with the method. Verbal probing was also used, to further understand any problems indicated during ‘think aloud’ method.^44,45^ Data was analysed using framework analysis.

### Phase IV: Initial psychometric validation (currently underway)

vi. Initial psychometric validation study, to ensure robust psychometric properties, comprising validity, reliability, responsiveness, interpretability and measure burden.^32–34^ This comprises a repeated observational questionnaire study with children with any life-limiting condition (aged 5–17), and/or parents/carers of a child <18 years old with a life-limiting condition, who complete C-POS:UK alongside several secondary measures. Additionally, health and social care professionals complete a clinician-proxy version of C-POS:UK.

## Ethics

Ethical considerations for each study are described in full elsewhere.^35,36,38,39^ The ethical approvals granted for each study are as follows: objective (i) granted by Bloomsbury research ethics committee (HRA:19/LO/0033); objectives (iii)–(iv) granted by King’s College London (MRSP-19/20-18826); objective (v) granted by the Bloomsbury research ethics committee (HRA: 21/LO/0282); objective (vi) granted by the Brighton and Sussex research ethics committee (HRA: 22/LO/0684).

In addition to formal ethical approvals, the C-POS:UK study benefits from the expert review of an external ethics advisor. This periodic appraisal has ensured support and transparency in the handling of any ethical matters raised by the project.

## Results

### Phase I: Gathering input on measure concept and design

i. A total of 106 participants: 26 children (5–17 years old), 40 parents, 13 siblings, 15 health and social care professionals and 12 commissioners participated in qualitative interviews. Diagnoses and ages of the children represented across the 79 child and family interviews were varied (6 children with cancer, 73 with non-cancer conditions; aged 0–17 years).

Stakeholders discussed matters across all domains of the WHO model of palliative care as relevant to children with life-limiting conditions. Challenging physical symptoms such as pain were commonly discussed. Spiritual and existential matters included a personal search for meaning behind illness, as well as the importance of planning for the future and leaving a legacy.^46^ Emotional and psychological concerns, were ubiquitous and included an awareness of differences with other children, and negative feelings such as fear, anger and sadness. Children and families are also worried about protecting one another from their own negative emotions. Social and practical concerns were characterised by a desire to enable children to engage in their usual activities, stay connected to friends and family, and where possible access appropriate education. Practical aspects of care, such as service availability, advanced care planning and the logistical challenges of managing care amongst other responsibilities were unique to adult participants. Children were shielded from these. A cross-cutting theme of pursuing normality was identified, which pertains to children’s desire to live as normally as possible.^35^

Overall 79 individuals contributed information within the qualitative interviews on measure design preferences of the target population, including 26 children with life-limiting or life-threatening conditions, 13 siblings and 40 parents.^38^ They addressed several important aspects of measure design. Children stated that measures that were brief in length were more feasible to use, while shorter recall periods of up to a week were considered more relevant to their needs. The use of response scales was feasible for children with life-limiting/life-threatening conditions, while scales with visual appeal made scales more relevant and acceptable. The majority of parents felt an online measure was most feasible and acceptable, but some children indicated a strong preference for a paper version of the PCOM. Children wanted to complete measures with a healthcare professional, as they valued the opportunity to talk about their responses.^38^

ii. A total of 81 articles met the inclusion criteria for the systematic review of the design of PROMs for children. Results demonstrated that relevant, comprehensible and feasible measures for children would require different versions for children depending on developmental stage and cognitive ability. Self-report was not deemed feasible for children under 5 years, thus clarifying the cut-off for proxy measure development. Unclear evidence meant it was difficult to establish feasible recall period and response format for children over 7. While the evidence was limited, children expressed a preference for computerised administration.^37^

Eight recommendations were made to support the development of measures for children: (1) proxy measures should be used for those under 5 years old; (2) measures should be visually appealing, to improve acceptability; (3) PROM studies should be analysed and reported in developmentally appropriate age bands; (4) developers should consider different versions of a measure for different age groups; (5) development should include both cognitive interview studies and psychometric testing to enhance understanding of how children formulate answers; (6) 5–7 years olds should be given a dichotomous response format, while those 7 years and over should be given a three-point response format; (7) recall period should be kept short, no more than 48 h for those 5–7 years; (8) PROMs should have a computerised version.^37^

The systematic review provided some clear guidance for C-POS:UK, and also some areas to consider carefully for the target population, through further input gathering, item generation and improvement phase.

### Phase II: Item generation

iii. The first round of the Modified ranking-style Delphi survey was completed by 82 participants (59 healthcare professionals, 23 parents/carers). The diagnoses represented by the parent/carer participants varied (1 child with cancer, and 22 with non-cancer conditions) and age (1–17 years old). The second round was completed by 60 participants (47 healthcare professionals, 13 parents/carers), and 30 participants (26 healthcare professionals, 4 parents/carers) completed the final round.

Although with each round agreement increased from weak to moderate, pre-defined consensus criteria (*K* > 0.7) was not reached. The study team decided to stop due to attrition, partly attributed to a challenging period comprising two national COVID-19 lockdowns, which increased the burden on expert participants.^47^ Although there were many similarities between parent/carer and professional rankings, parents prioritised psychosocial concerns and physical functioning of their child, while professionals prioritised physical symptoms (e.g. pain).^36^

COSMIN standards highlight the need to involve the target population through appropriate methods.^24^ Within the YPAG there were similarities with the adult rankings, but young people had some different priorities. These differences pertained to living a ‘normal life’ (e.g. maintaining relationships with peers, and accessing education), which informed one item.^36^ Visual appeal was identified as an important aspect to support relevance and acceptability in phase I. In response, the YPAG was invited to co-design C-POS:UK. They selected planets as labels to be used instead of chronological age, which adds visual appeal. It also identifies the different versions of C-POS:UK minus any stigma associated with a lack of concordance between a child’s age and developmental stage.

iv. There were 22 participants in the item generation meeting, including nine paediatric palliative healthcare professionals, six research team members, five clinical academics with expertise in PCOM development and two parents bereaved of a child with a life-limiting condition. The meeting began with presentation of the results of the four robust sequential studies described above (objectives (i)–(iv)), guided by COSMIN and Rothrock standards. Presentations focussed on the details of the construct to be measured by C-POS:UK and related domains (objective (i) results), measure design (objectives (ii) and (iii) results) and priority items for inclusion (objective (iv) results). Suggested item wording was presented and discussed.^36^

It was challenging to develop a measure that would be relevant and comprehensive for all children and young people across a range of life-limiting and threatening conditions. Pragmatic decisions about how to be inclusive therefore had to be taken – for example a generic item was developed to cover symptoms other than pain.

A total of five versions of C-POS:UK were agreed upon. There are three versions that allow children of different ages/developmental phases to self-report their own outcomes and two proxy-report versions for parent/carer completion when the child cannot participate. Multiple versions were necessary for comprehensibility, relevance and feasibility. Every version consists of eight items relating to child outcomes, which cover the domains identified in the qualitative interview study (objective (i)). These domains are emotional and psychological concerns, physical symptoms, practical aspects of care, and spiritual and existential matters (see Table 1 for further details on items). The two parent/carer versions include proxy questions to establish child outcomes where the child cannot participate themselves.^36^ The item generation meeting also established that each version of C-POS:UK was to include five proxy-reported items focussed on the family which the parent/carer answers. These items also cover the aforementioned domains, but the focus is on family concerns. This inclusion is vital, given the central role that families have in their child’s palliative care.

**Table 1.** C-POS:UK domains and item topics*, as established through item generation and item improvement phases.

Domain	Question item		
	As established in phase II – item generation^36^	As established by end of phase III – cognitive interview study^39^	
	Self/proxy-reported	Self-reported	Proxy-reported
Child item topics*			
Physical	Pain	Hurt (cognitive ability of 5–12 years) Pain (cognitive ability of 13–17 years)	Pain
	Other symptoms	Other problems with your body	Other symptoms
Social and practical	Being able to ask questions	Being able to ask important questions	Communicate needs (for proxy for child <2 years) Had the appropriate information for them about their condition (for proxy for child >2 years)
	Being able to undertake usual activities	Being able to do the things you usually would	Being able to do the things child usually would
Emotional and psychological	Worry	Worry	Displayed signs of worry or anxiety for example, by being more irritable, sad, clingy or withdrawn (for proxy for child <2 years) Expressed anxiety and worry (for proxy for child >2 years)
	Sharing feelings	Being able to talk to people (cognitive ability of 5–7 years) Sharing feelings (cognitive ability of 8–17 years)	Express feelings (for proxy for child <2 years) Opportunity to express feelings (for proxy for child >2 years)
	Being able to do things you enjoy	Being able to do things that are fun (cognitive ability of 5–7 years) Being able to do things you enjoy (cognitive ability of 8–17 years)	Being able to do things child enjoys
Spiritual and existential	Being able to do things you enjoy	*[as above]*	*[as above]*
	Living life to the fullest	Enjoying life as much as possible (cognitive ability of 5–12 years) Living life to the fullest (cognitive ability of 13–17 years)	Live life to *their* fullest
Family item topics (proxy-reported)*			
Physical	Getting enough sleep	Able to get enough sleep	
Social and practical	Access to information about child’s condition	Information about child’s condition	
	Support needed to care for child	Support needed to provide care	
	Support to plan future care	*[as below]*	
Emotional and psychological	Impact of child’s condition on family	Impact of child’s condition on family	
		*[NEW ITEM]* Access to psychological and emotional support	
Spiritual and existential	Support to plan future care	Planning for future care	

Source: This table has been adapted from Coombes et al.^36^ and Coombes et al.,^39^ both licensed under a Creative Commons Attribution International license (CC BY 4.0).

* Please note the item topics as presented in this table are not exact item wording and should not be used in practice. C-POS:UK is currently undergoing psychometric validation, and the final wording and item order will be published in due course.

### Phase III: Item improvement

v. The five versions of C-POS:UK, developed through item generation, were cognitively tested with a total of 48 participants, comprising 12 children with a life-limiting condition and 36 parents.^39^ Diagnoses of the children represented across the 48 interviews were diverse (8 children with cancer, 40 with non-cancer conditions).

Between two and seven rounds of cognitive interviews were conducted, and each of the final versions was tested in their final format, as proposed by COSMIN guidelines for content validity.^24^ Although some parents highlighted that completing a PCOM on this topic might be distressing, the study confirmed that it was important to be asked, and that the content and length of C-POS:UK was acceptable for the target population.

Data on recall period demonstrated that the C-POS:UK version for less cognitively able requires a short recall period of ‘yesterday and today’, while all other groups can use a recall of the ‘past week’. A three-point Likert scale was suitable for children across two versions (covering cognitive ages of 5–7 years and 8–12 years), while both parent/carer versions and the third child version (cognitive age 13–17 years) worked best with a five-point Likert scale. The wording of some items was improved to rectify problems with comprehension.

Minor but important changes were made to ensure relevance for families of children who are non-verbal.

There were no suggestions for additional items from children. However, parents noted the need for an additional item on psycho-emotional support for themselves and other family members. This was subsequently added, and so increased the number of items focussed on family concerns to six.

Adaptations have ensured that the five versions of C-POS:UK are comprehensive, comprehensible and relevant for children living with a wide variety of life-limiting conditions, and their families, and therefore ready for psychometric testing.

### Phase IV: Initial psychometric validation (currently underway)

vi. Children with life-limiting conditions, their parents/carers and professionals who care for them have completed the self-report questionnaire. A study to evaluate the psychometric properties of C-POS:UK is currently underway, with support from sites across the four nations of the UK: England, Scotland, Wales and Northern Ireland.

## Expert stakeholder involvement

Successful research requires the involvement of people with relevant experience to help shape and direct the project. Throughout this sequential mixed-method study, guided by Rothrock et al. and COSMIN, we have actively engaged with expert stakeholders, to inform and improve the development and testing of C-POS:UK.

## Cross-national multi-disciplinary steering group

The multi-disciplinary expert C-POS:UK steering group includes paediatric palliative care health and social care professionals, key advocacy groups, clinical academics, academics and researchers who specialise in palliative care, outcome measure development, ethics, and qualitative and quantitative methods. Through regular steering group meetings throughout the project, this rich and diverse group has informed key decision-making. They have advised on refinements in methods, recruitment processes and engagement, and have been central in supporting improvements in the content validity, feasibility and acceptability of C-POS:UK. This work is continuing through the ongoing psychometric validation.

## Patient and public involvement

Some children with life-limiting conditions are experts in their condition.^48^ They have the right to express their views and have them taken seriously in all matters affecting them.^49^ This includes their priorities for their care. Children with life-limiting conditions have been directly involved in the C-POS:UK studies wherever possible (objectives (i), (iii), (v) and (vi)). We have bolstered children’s involvement by working with an existing YPAG, run by a UK children’s hospital, throughout the project.^50–52^ This has helped to ensure that children’s views are represented in a meaningful way throughout the development and refinement of C-POS:UK. The young people in this group are aged 10–21 years old and comprise children with a life-limiting condition, siblings of children with a life-limiting condition, and children with aspirations to work in healthcare or research. The YPAG has provided important input on several aspects of C-POS:UK design, including optimal recall period, response format and the use of emojis to anchor response scales. They have also informed decisions on appropriate wording for children’s participant information sheets and study instructions. This has strengthened study documentation and processes.

Many children with life-limiting conditions are either too young or have developmental delays severe enough that they cannot contribute meaningfully to patient and public involvement (PPI) involvement in research studies or complete outcome measures themselves. Parental views are vital for this group. We have ensured that parent perspectives were included through participation in the sequential studies (objectives (i), (iii), (iv), (v) and (vi)), and with the involvement of three parents bereaved of a child with a life-limiting condition. The bereaved parent PPI members are vital to steering group meetings, advising at all stages of the study and supporting decisions using their expertise and unique perspectives. They have been instrumental in ensuring a proportionate and sensitive approach to research with children with life-limiting conditions and their families. They have supported key ethical decisions, attended and contributed to ethical review meetings, and provided input and statements for important ethical amendments. A blog from the three bereaved parent PPI members demonstrates the importance of involving these experts in C-POS:UK development and validation work.^53^

We continue to involve children and parent PPI members in this advisory capacity as we progress through the psychometric validation phase, and towards implementation of C-POS:UK.

## Discussion

Building on a review that demonstrated the absence of an appropriate outcome measure for children with life-limiting conditions,^16^ investigation progressed to the expert stakeholder workshop.^13^ These pre-development studies elucidated the need and support for such a measure across healthcare, research and advocacy groups, and thus laid the foundation for this sequential mixed-methods approach to developing and validating C-POS:UK.

Qualitative interviews allowed us to gain an in-depth understanding of the priorities for care for children with life-limiting illness and their families,^35^ and specific measure design requirements within the target population.^38^ The systematic review elucidated best practices in self-reported outcome measures designed for children.^37^ The consensus study and YPAG consultation clarified priority items from a list of potential items developed using qualitative interview data. All of the data collected prior then fed into the subsequent expert item generation meeting, which facilitated drafting C-POS:UK.^36^ This initial child and family centred outcome measure was cognitively tested and refined.^39^ The robust output from this work was the first version of C-POS:UK, comprising three child self-report versions (i.e. Mercury, Saturn, Neptune, as selected by the YPAG) and two parent/carer proxy versions. The refined C-POS:UK is now undergoing psychometric validation. Working closely with the multi-disciplinary expert steering group and PPI contributors throughout each stage has strengthened research processes and outputs.

## Conclusion

The C-POS:UK studies have followed a novel approach, guided by a combination of the Rothrock measure development process and COSMIN standards. This careful and robust sequential work has advanced the science and practice of outcomes measurement in children’s palliative care, with strong child and parent input in the decision-making. The next steps will be to share the findings of the validation before moving into the implementation phases.

## Supplemental Material

sj-docx-1-pcr-10.1177_26323524241303537 – Supplemental material for Development of a child and family centred outcome measure for children and young people with life-limiting and life-threatening conditions: progress to date on the Children’s Palliative Care Outcome Scale (C-POS:UK) Supplemental material, sj-docx-1-pcr-10.1177_26323524241303537 for Development of a child and family centred outcome measure for children and young people with life-limiting and life-threatening conditions: progress to date on the Children’s Palliative Care Outcome Scale (C-POS:UK) by Debbie Braybrook, Lucy Coombes, Daney Harðardóttir, Hannah M. Scott, Katherine Bristowe, Clare Ellis-Smith, Anna Roach, Christina Ramsenthaler, Myra Bluebond-Langner, Julia Downing, Fliss E. M. Murtagh, Lorna K. Fraser and Richard Harding in Palliative Care and Social Practice

## Declarations

**Ethics approval and consent to participate:** The ethical approvals granted for each study are as follows: objective (i) granted by Bloomsbury research ethics committee (HRA:19/LO/0033); objectives (iii)–(iv) granted by King’s College London (MRSP-19/20-18826); objective (v) granted by the Bloomsbury research ethics committee (HRA: 21/LO/0282); objective (vi) granted by the Brighton and Sussex research ethics committee (HRA: 22/LO/0684). Consent to participate and for publication of each study is as follows: objective (i) Participants over 16 years old provided written informed consent, participants with parental responsibility provided written informed consent for participants under 16 years, and participants under 16 years provided written assent; objectives (iii)–(iv) Participants received written study information and completed a consent form at the beginning of each round; objective (v) Participants over 16 years provided written informed consent, participants with parental responsibility provided written informed consent for participants under 16 years, and participants under 16 years were given the opportunity to provide written assent; objective (vi) Participants over 16 years old provided written informed consent, participants with parental responsibility provided written informed consent for participants under 16 years, and participants under 16 years provided written assent. For young people aged 16–17 without sufficient capacity to consent, a personal consultee was identified to advise on the young person’s wishes regarding participation.

**Consent for publication:** Informed consent for publication was provided by participants, or an authorised representative, in line with the abovementioned consent processes.

**Availability of data and materials:** It is not possible for the data from this study to be made available.
