# Supplementary material for: Development of a child and family centred outcome measure for children and young people with life-limiting and life-threatening conditions: progress to date on the Children’s Palliative Care Outcome Scale (C-POS:UK)
Source: Palliat Care Soc Pract. 2024 Dec 17;18:26323524241303537. doi: 10.1177/26323524241303537 (PMC11650473; doi:10.1177/26323524241303537)
Supplement: sj-docx-1-pcr-10.1177_26323524241303537 – Supplemental material for Development of a child and family centred outcome measure for children and young people with life-limiting and life-threatening conditions: progress to date on the Children’s Palliative Care Outcome Scale (C-POS:UK) [file sj-docx-1-pcr-10.1177_26323524241303537.docx]

**Good Reporting of A Mixed Methods Study (GRAMMS)**

| **Item** | **Guideline** | **Reported on page no**. |
| --- | --- | --- |
| **(1)** | Describe the justification for using a mixed methods approach to the research question | Page 4 |
| **(2)** | Describe the design in terms of the purpose, priority and sequence of methods | Pages 3-6, and illustrated in figure 1 |
| **(3)** | Describe each method in terms of sampling, data collection and analysis | Pages 5-6 |
| **(4)** | Describe where integration has occurred, how it has occurred and who has participated in it | Page 5 for integration of data to support objective iii; page 6 for objective ii, iv and v. |
| **(5)** | Describe any limitation of one method associated with the presence of the other method | Page 6 for children’s engagement in item generation (objective iii). |
| **(6)** | Describe any insights gained from mixing or integrating methods | Pages 7-11 |

Developed from: O’Cathain A, Murphy E, Nicholl J. The Quality of Mixed Methods Studies in Health Services Research. *Journal of Health Services Research & Policy*. 2008;13(2):92-98. doi:10.1258/jhsrp.2007.007074
